# Prognostic impact of the C-reactive protein–albumin–lymphocyte index in patients with resected lung cancer associated with interstitial pneumonia

**DOI:** 10.1007/s00595-026-03250-y

**Published:** 2026-03-14

**Authors:** Chihiro Konoeda, Gouji Toyokawa, Mototsugu Shimokawa, Masaaki Sato

**Affiliations:** 1 Department of Thoracic Surgery, The University of Tokyo Hospital, Tokyo, Japan; 2 Department of Surgery and Science, Graduate School of Medical Sciences, Kyushu University, Fukuoka, Japan; 3 Department of Biostatistics, Graduate School of Medicine, Yamaguchi University, 7-3-1 Hongo, Bunkyo-Ku, Tokyo, 113-8655 Japan

**Keywords:** Lung cancer, Interstitial pneumonia, C-reactive protein–albumin–lymphocyte index, Surgery, Prognosis

## Abstract

**Purpose:**

The C-reactive protein (CRP)–albumin–lymphocyte (CALLY) index is a composite score that evaluates inflammatory and nutritional status. This study aimed to determine its prognostic significance in patients with resected lung cancer associated with interstitial pneumonia (IP).

**Methods:**

Eighty-five patients with IP-associated resected lung cancers were included. The CALLY index was calculated as follows: (serum albumin × lymphocytes) / (CRP × 10^4^). The patients were classified into low and high CALLY groups based on a cutoff value determined by the receiver operating characteristic curve for overall survival (OS). The associations between the CALLY index, patient characteristics, and postoperative survival were retrospectively investigated.

**Results:**

The postoperative hospital stay was significantly longer (*P* = 0.015) and the incidence of postoperative pneumonia was significantly higher (*P* = 0.004) in the low CALLY group (n = 20, 23.5%) than in the high CALLY group (n = 65, 82.5%). The recurrence-free survival (RFS) and OS were significantly poorer in the low CALLY group (*P* = 0.045 and* P* = 0.020, respectively). A multivariate analysis identified the CALLY index as an independent prognosticator factor for RFS and OS (*P* = 0.013 and* P* = 0.004, respectively).

**Conclusion:**

The CALLY index is an independent prognostic factor in patients with IP-associated resected lung cancer.

**Supplementary Information:**

The online version contains supplementary material available at 10.1007/s00595-026-03250-y.

## Introduction

Patients with interstitial pneumonia (IP) are at a high risk of developing lung cancer, and IP-associated lung cancer is biologically more aggressive than lung cancer without IP [1]. Several studies have demonstrated that the postoperative survival outcomes are worse in patients with IP associated with lung cancer than in those without IP [2–5]. Despite the pivotal role of surgery in the treatment of IP-associated lung cancer, a major concern during the perioperative period is acute exacerbation of IP (IP-AE), which has an incidence of 9.3% within 30 days of pulmonary resection and a mortality of 43.9% [6, 7]. Furthermore, patients with IP-associated lung cancer tend to be older and have a more frequent smoking history than those without IP, indicating that attention should also be paid to perioperative complications other than IP-AE [2, 3].

The C-reactive protein (CRP)–albumin–lymphocyte (CALLY) index, derived from serum albumin, lymphocyte count, and CRP, was originally developed as a biomarker for predicting the prognosis after hepatectomy in patients with hepatocellular carcinoma [8]. It is based on a design that combines the prognostic nutritional index (PNI), derived from serum albumin and lymphocyte counts, and the modified Glasgow prognostic score (mGPS), derived from serum albumin and CRP levels [9, 10]. Subsequent reports have demonstrated that the CALLY index could also serve as a prognostic indicator in resected esophageal squamous cell carcinoma, gastric cancer, breast cancer, and non-small cell lung cancer [11–14]. The CALLY index has also been shown to predict the prognosis of patients with diseases other than cancer, including infectious diseases and acute ischemic stroke [15–17]. Importantly, Xu et al. demonstrated the usefulness of the CALLY index in predicting postoperative pneumonia in esophageal cancer [18]. However, the significance of the CALLY index is yet to be investigated in patients with resected lung cancer associated with IP.

The present study investigated the associations between the CALLY index, clinicopathological features, and perioperative outcomes, including IP-AE, pneumonia, and postoperative survival, in patients with resected lung cancer associated with IP.

## Methods

### Study cohort

Eighty-seven of 1,346 patients who underwent surgery for lung cancer at The University of Tokyo Hospital between January 2011 and March 2021 were retrospectively diagnosed with IP. Two patients were excluded because of insufficient data on the CALLY index, leaving 85 patients for inclusion in the study. Information was collected on clinicopathological characteristics, including age at surgery, sex, body mass index, smoking history, respiratory function, radiological findings, clinical and pathological tumor–node–metastasis (TNM) stage (according to the 7th edition of the lung cancer staging system), surgical procedure, type of histology, and vessel, lymphatic, and pleural invasion status. Perioperative variables included postoperative hospital stay, operation time, blood loss, need for re-operation, cardiovascular complications, bronchopulmonary fistula, pneumonia, empyema, prolonged air leak, IP-AE, and death during the postoperative hospital stay. After pulmonary resection, the routine follow-up consisted of physical examinations, blood tests, and chest radiographs, which were performed every 3 months for the first 2–3 years and 6-monthly thereafter. Computed tomography (CT) scans were performed twice yearly during the first 5 years and at least annually thereafter for up to 10 years after surgery. Follow-up data were collected until March, 2025. This study was approved by the Ethics Committee of the University of Tokyo Hospital (IRB approval number 2406–[11]). The need for informed consent was waived owing to the retrospective observational nature of the study. However, patients were given the opportunity to withhold their personal data via the opt-out route.

### Diagnosis of IP and AE

IP was diagnosed by multidisciplinary discussions of the physical, serological, high-resolution CT, and pathological findings after surgical resection [19, 20]. Other pulmonary diseases were excluded by examination of the resected lung specimens. Postoperative IP-AE following lung resection was defined based on previous reports [21]. Data on postoperative IP-AE and IP-AE risk scores were also collected [22]. The GAP score was used as an indicator of IP severity [23].

### Calculation of CRP, albumin, the CALLY index, PNI, and mGPS

Blood tests, including serum albumin, lymphocyte count, and CRP, were obtained during the month before surgery. In accordance with a previous report, the CALLY index was calculated using the following formula: (albumin [g/dL] × lymphocyte count [/μL]) / (CRP [mg/dL] × 10^4^) [8]. The PNI was calculated as follows: 10 × serum albumin (g/dL) + 0.005 × lymphocyte count (/μL) [9]. The mGPS was defined using the following method: a score of 2 for both CRP > 0.5 mg/dL and albumin < 3.5 g/dL; a score of 1 for CRP > 0.5 mg/dL and albumin ≥ 3.5 g/dL/CRP ≤ 0.5 mg/dL and albumin < 3.5 g/dL; or a score of 0 for both CRP ≤ 0.5 mg/dL and albumin ≥ 3.5 g/dL [10]. The cutoff values for CRP, albumin, CALLY index, and PNI were determined by analysis of the receiver operating characteristic (ROC) curve for postoperative overall survival (OS). The patients were divided into a group with an mGPS of 0 and a group with an mGPS of 1 or 2.

### Statistical analysis

Categorical variables were summarized as numbers and/or percentages, and continuous variables as medians (interquartile range [IQR]). The recurrence-free survival (RFS) was defined as the interval between the date of surgery and the date of recurrence, death, or last follow-up. OS was defined as the interval between the date of surgery and the date of death from any cause or the last follow-up. Associations between the groups and continuous variables were analyzed using the Mann–Whitney U test, and associations with categorical variables were evaluated using the chi-square test. Survival probabilities were estimated using the Kaplan–Meier method, and differences between survival curves were assessed using the log-rank test. The risk factors for RFS and OS were assessed using a Cox proportional hazards model with forced entry. Preoperative variables, including age, sex, clinical stage, and the CALLY index, were included in the multivariate analysis. Missing data were not included. All analyses were performed using JMP® 18.0 (SAS Institute Inc., Cary, NC, USA) and Prism 8.0 (GraphPad Software Inc., San Diego, CA, USA). A *P*-value of < 0.05 was considered to be statistically significant.

## Results

### Patient characteristics

Table 1 shows the the patient characteristics. There were 20 women (23.5%) and 65 men (76.5%), with a median age of 73 years [IQR 66, 78]. Seventy-four (87.1%) patients had a history of smoking. Fifteen patients (17.6%) had a preoperative history of steroid use. The median percentage vital capacity (%VC) was 96.0% [IQR 85.5, 108.7], with 13 patients (15.3%) having a %VC of less than 80%. Radiologically, the usual IP pattern was observed in 34 patients (40.0%). Seventy (82.4%) and 50 (58.8%) patients had clinical and pathological stage I diseases, respectively. A sublobar resection was performed in 21 (24.8%) patients. Forty-three patients (50.6%) had adenocarcinoma and 28 (32.9%) had squamous cell carcinoma. Forty-five patients experienced recurrence and 45 (52.9%) died after surgery. The distribution of the GAP scores was as follows: 25 patients (30.1%) had scores of 0 or 1, 52 patients (62.7%) had scores of 2 or 3, and 6 patients (7.2%) had scores of 4 or 5.

**Table 1:** Demographic and clinicopathological characteristics of the 85 patients with resected lung cancer associated with interstitial pneumonia

Characteristics		N = 85
Age (years)	Median (IQR)	73 (66, 78)
Sex	Female	20 (23.5%)
	Male	65 (76.5%)
BMI (kg/m^2^)	Median (IQR)	23.0 (21.0, 25.0)
Smoking history	Never	11 (12.9%)
	Ex	48 (56.5%)
	Current	26 (30.6%)
Brinkman index	Median (IQR)	900 (650, 1380)
Preoperative steroids	No	70 (82.4%)
	Yes	15 (17.6%)
History of IP-AE	No	85 (100.0%)
	Yes	0 (0.0%)
KL-6 (U/ml)*	Median (IQR)	554 (364, 907)
FEV1.0 (mL)	Median (IQR)	2230 (1830, 2650)
FEV1.0% (G)	Median (IQR)	75.0 (67.7, 79.1)
VC (mL)	Median (IQR)	3030 (2475, 3825)
%VC	Median (IQR)	96.0 (85.5, 108.7)
	> 80%	72 (84.7%)
	≤ 80%	13 (15.3%)
%DLCO’ (%)**	Median (IQR)	88.1 (76.5, 102.9)
	> 80%	55 (66.3%)
	≤ 80%	28 (33.7%)
Radiological findings of IP	UIP	34 (40.0%)
	Probable UIP	33 (38.8%)
	Indeterminate or alternative	18 (21.2%)
Clinical stage	0	0 (0.0%)
	I	70 (82.4%)
	II	9 (10.6%)
	III	6 (7.0%)
Surgical procedure	Wedge resection	19 (22.4%)
	Segmentectomy	2 (2.4%)
	Lobectomy or bilobectomy	64 (75.2%)
Histology	Adenocarcinoma	43 (50.6%)
	Squamous cell carcinoma	28 (32.9%)
	Others	14 (16.5%)
Pathological stage	0	2 (2.4%)
	I	50 (58.8%)
	II	14 (16.5%)
	III	19 (22.3%)
ly	Absent	49 (57.6%)
	Present	36 (42.4%)
v	Absent	28 (32.9%)
	Present	57 (67.1%)
pl	Absent	46 (54.1%)
	Present	39 (45.9%)
Recurrence	Absent	40 (47.1%)
	Present	45 (52.9%)
Postoperative survival	Alive	40 (47.1%)
	Dead	45 (52.9%)
GAP score**	0, 1	25 (30.1%)
	2, 3	52 (62.7%)
	4, 5	6 (7.2%)
	6–8	0 (0.0%)
CALLY index	Median (IQR)	3.98 (1.10, 10.93)

^*^ Data were missing for 4 patients

^**^Data were missing for 2 patients

AE, acute exacerbation; BMI, body mass index; CALLY, C-reactive protein–albumin–lymphocyte count; DLCO, diffusing capacity of the lung for carbon monoxide; FEV1.0, forced expiratory volume in 1 s; IP, interstitial pneumonia; IQR, interquartile range; KL-6, Krebs von den Lungen-6; UIP, usual interstitial pneumonia; VC, vital capacity

### Cut-off value of CRP, albumin, the CALLY index and the PNI

The median CALLY index value was 3.98 [IQR 1.10, 10.93] (Table 1). The ROC curve for OS after surgery revealed that the optimal cutoff value for the CALLY index was 0.97, with a sensitivity of 86.7%, specificity of 32.5%, and an area under the curve (AUC) of 0.577 (Supplementary Fig. 1). The cutoff PNI value was 43.94, with a sensitivity, specificity, and AUC of 75.6%, 42.5%, and 0.544, respectively (Supplementary Fig. 2a). The cut-off values for CRP and albumin were 0.07 and 4.0, with AUCs of 0.579 and 0.535, respectively (data not shown).

### Comparison of the patient characteristics and perioperative outcomes between the high and low CALLY groups

A %VC of less than 80% was significantly more common in the low CALLY group (*P* = 0.037), and the forced expiratory volume in 1 s % was significantly lower in the high CALLY group (*P* = 0.043) (Table 2). Preoperative steroid use was more common in the low CALLY group (*P* = 0.020). Additionally, the low CALLY group tended to have a higher frequency of vessel invasion than the high CALLY group (*P* = 0.051). No statistically significant difference was observed between the CALLY and GAP scores (*P* = 0.084). In terms of perioperative outcomes, the postoperative hospital stay was significantly longer in the low CALLY group (16 days [IQR 9, 30] vs. 10 days [IQR 7, 14], *P* = 0.015), and the incidence of postoperative pneumonia was significantly higher in this group (*P* = 0.004) (Table 3). The frequency of IP-AE was higher in the low CALLY group (*P* = 0.064).

**Table 2:** Comparison of the clinicopathological characteristics according to whether the CALLY index was low or high

Characteristics		Low CALLY (n = 20)	High CALLY (n = 65)	*P*-value
Age (years)	Median (IQR)	73 (62, 79)	72 (67, 77)	0.992
Sex	Female	6 (30.0%)	14 (21.5%)	0.435
	Male	14 (70.0%)	51 (78.5%)	
BMI (kg/m^2^)	Median (IQR)	23.0 (21.0, 26.2)	23.0 (21.0, 25.0)	0.896
Smoking history	Never	3 (15.0%)	8 (12.3%)	0.754
	Ex or current	17 (85.0%)	57 (87.7%)	
Brinkman index	Median (IQR)	875 (325, 1357)	900 (710, 1395)	0.507
Preoperative steroids	No	13 (65.0%)	57 (87.7%)	0.020
	Yes	7 (35.0%)	8 (12.3%)	
History of IP-AE	No	20 (100.0%)	65 (100.0%)	-
	Yes	0 (0.0%)	0 (0.0%)	
KL-6 (U/ml)*	Median (IQR)	677 (506, 1013)	506 (348, 892)	0.209
	≤ 1000	14 (77.8%)	51 (81.0%)	0.765
	> 1000	4 (22.2%)	12 (19.0%)	
FEV1.0 (mL)	Median (IQR)	2175 (1820, 2515)	2240 (1830, 2680)	0.675
FEV1.0% (G)	Median (IQR)	78.5 (70.6, 81.1)	72.0 (67.2, 78.3)	0.043
VC (mL)	Median (IQR)	2870 (2220, 3290)	3230 (2525, 3845)	0.153
%VC (%)	Median (IQR)	87.5 (66.0, 117.4)	97.5 (88.0, 107.0)	0.257
	> 80%	14 (70.0%)	58 (89.2%)	0.037
	≤ 80%	6 (30.0%)	7 (10.8%)	
%DLCO’ (%)**	Median (IQR)	83.8 (74.7, 98.4)	88.3 (76.5, 104.6)	0.580
	> 80%	13 (65.0%)	42 (66.7%)	0.891
	≤ 80%	7 (35.0%)	21 (33.3%)	
Radiological findings of IP	UIP	4 (20.0%)	14 (21.5%)	0.804
	Probable UIP	9 (45.0%)	24 (36.9%)	
	Indeterminate or alternative	7 (35.0%)	27 (41.6%)	
Clinical stage	0 or I	16 (80.0%)	54 (83.1%)	0.752
	≥ II	4 (20.0%)	11 (16.9%)	
Surgical procedure	Sublobar resection	4 (20.0%)	17 (26.2%)	0.577
	Lobectomy or bilobectomy	16 (80.0%)	48 (73.8%)	
Histology	Adenocarcinoma	9 (45.0%)	34 (52.3%)	0.500
	Squamous cell carcinoma	6 (30.0%)	22 (33.9%)	
	Others	5 (25.0%)	9 (13.9%)	
Pathological stage	0 or I	11 (55.0%)	41 (63.1%)	0.517
	≥ II	9 (45.0%)	24 (36.9%)	
ly	Absent	13 (65.0%)	36 (55.4%)	0.447
	Present	7 (35.0%)	29 (44.6%)	
v	Absent	3 (15.0%)	25 (38.5%)	0.051
	Present	17 (85.0%)	40 (61.5%)	
pl	Absent	10 (50.0%)	36 (55.4%)	0.673
	Present	10 (50.0%)	29 (44.6%)	
GAP score**	0, 1	10 (50.0%)	15 (23.8%)	0.084
	2, 3	9 (45.0%)	43 (68.3%)	
	4, 5	1 (5.0%)	5 (7.9%)	

^*^ Data were missing for 4 patients

^**^Data were missing for 2 patients

AE, acute exacerbation; BMI, body mass index; CALLY, C-reactive protein–albumin–lymphocyte count; DLCO, diffusing capacity of the lung for carbon monoxide; FEV1.0, forced expiratory volume in 1 s; IP, interstitial pneumonia; IQR, interquartile range; KL-6, Krebs von den Lungen-6; UIP, usual interstitial pneumonia; VC, vital capacity

**Table 3:** Comparison of the perioperative outcomes according to whether the CALLY index was low or high

Characteristics		Low CALLY (n = 20)	High CALLY (n = 65)	*P*-value
Postoperative hospital stay (days)	Median (IQR)	16 (9, 30)	10 (7, 14)	0.015
Operation time (min)	Median (IQR)	201 (131, 257)	178 (117, 240)	0.433
Blood loss (mL)	Median (IQR)	125 (12, 272)	100 (5, 180)	0.445
Re-operation	Absent	19 (95.0%)	61 (93.8%)	0.848
	Present	1 (5.0%)	4 (6.2%)	
Cardiovascular complications	Absent	19 (95.0%)	64 (98.5%)	0.372
	Present	1 (5.0%)	1 (1.5%)	
Bronchopulmonary fistula	Absent	20 (100.0%)	64 (98.5%)	0.577
	Present	0 (0.0%)	1 (1.5%)	
Pneumonia	Absent	14 (70.0%)	61 (93.8%)	0.004
	Present	6 (30.0%)	4 (6.2%)	
Empyema	Absent	19 (95.0%)	62 (95.4%)	0.943
	Present	1 (5.0%)	3 (4.6%)	
Prolonged air leak	Absent	16 (80.0%)	56 (86.2%)	0.504
	Present	4 (20.0%)	9 (13.8%)	
AE of IP	Absent	16 (80.0%)	61 (93.9%)	0.064
	Present	4 (20.0%)	4 (6.1%)	
Postoperative death during the hospital stay	Absent	19 (95.0%)	64 (98.5%)	0.372
	Present	1 (5.0%)	1 (1.5%)	

AE, acute exacerbation; CALLY, C-reactive protein–albumin–lymphocyte count; IP, interstitial pneumonia; IQR, interquartile range

### Analysis of risk factors for postoperative survivals

**Fig. 1:**
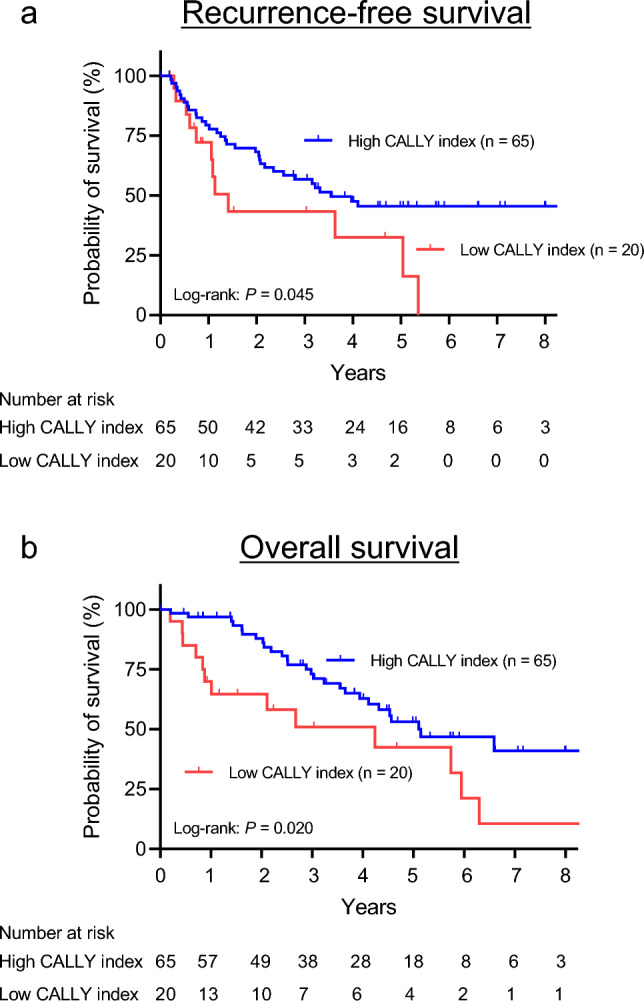
Survival following surgery in patients with lung cancer associated with IP. Comparison of (a) the recurrence-free survival and (b) the overall survival between the low and high CALLY groups. CALLY, C-reactive protein–albumin–lymphocyte count

The median follow-up was 1,112 days. The median RFS and OS were significantly worse in the low CALLY group (515 vs. 1,294 days for RFS, *P* = 0.045; 1,546 vs. 1,862 days for OS, *P* = 0.020) (Fig. 1). There was no significant difference in OS between the high and low PNI groups (*P* = 0.099) (Supplementary Fig. 2b) or between the group with mGPS 0 and the group with mGPS 1 or 2 (*P* = 0.110) (Supplementary Fig. 2c). No significant difference was observed in OS between the high and low CRP groups (P = 0.279) (Supplementary Fig. 3a) or between the high and low albumin groups (P = 0.321) (Supplementary Fig. 3b). A multivariate analysis identified age as a significant independent prognostic factor for RFS (hazard ratio [HR] 2.954, 95% confidence interval [CI] 1.274–6.850; *P* = 0.012), cStage (HR 3.463, 95% CI 1.192–210.065; *P* = 0.023), and CALLY index (HR 2.955, 95% CI 1.253–6.967; *P* = 0.013) (Table 4). For OS, cStage (HR 2.518, 95% CI, 1.125–5.634; *P* = 0.025) and CALLY index (HR 2.901, 95%CI, 1.411–5.968; *P* = 0.004) were significant independent prognostic factors.

**Table 4:** Results of multivariate analyses of the RFS and OS in patients with resected lung cancer associated with interstitial pneumonia

Variables	RFS				**OS**		
	HR	95% CI	*P *value		HR	95%CI	*P* value
Age (≥ 70 years/ < 70 years)	2.954	1.274–6.850	0.012		1.112	0.540–2.289	0.774
Sex (male/female)	0.777	0.351–1.718	0.533		2.478	0.961–6.388	0.060
cStage (≥ II/I)	3.463	1.192–10.065	0.023		2.518	1.125–5.634	0.025
CALLY index (low [≤ 0.97]/high [> 0.97])	2.955	1.253–6.967	0.013		2.901	1.411–5.967	0.004

CALLY, C-reactive protein–albumin–lymphocyte count; CI, confidence interval; HR, hazard ratio; OS, overall survival; RFS, recurrence-free survival

## Discussion

In this study of patients with resected lung cancer associated with IP, the low CALLY group was significantly associated with several clinicopathological characteristics and perioperative outcomes, including a %VC lower than 80%, prolonged postoperative hospital stay, and a higher incidence of postoperative pneumonia, compared to the high CALLY group. Furthermore, the CALLY index significantly stratified both the RFS and OS. These findings align with previous reports showing associations between the CALLY index and postoperative pneumonia in esophageal cancer [18] and postoperative prognosis in several types of cancer [11–14]. Importantly, our results indicate that the CALLY index may be useful for stratifying the postoperative risk in such patients. This index is calculated using readily available variables, including serum albumin, lymphocyte count, and CRP, and can be easily and inexpensively applied in clinical settings. Moreover, patients with a low CALLY index might benefit from preoperative interventions, such as anamorelin [24], a ghrelin receptor agonist approved for the treatment of cachexia, characterized by weight loss in certain types of cancer, to improve their nutritional status. Nerandomilast, a phosphodiesterase 4 B inhibitor with antifibrotic and immunomodulatory effects, is also expected to improve immune competence and the inflammatory response [25].

As defined by Iida et al., the CALLY index includes serum albumin, lymphocyte count, and CRP and can reflect the status of nutrition, the immune system, and inflammation [8]. Their design for establishing the CALLY index was based on combining the PNI and mGPS, and the superiority of the CALLY index over the PNI was demonstrated in predicting the prognosis after hepatectomy for hepatocellular carcinoma. In the present study, there was no significant difference in OS between the high and low PNI groups, and the ROC-AUC of the PNI for predicting OS was lower than that of the CALLY index. Furthermore, there was no significant difference in the OS between the mGPS 0 and mGPS 1 or 2 groups. Additionally, no significant difference was observed in the OS based on CRP or albumin levels. These findings highlight the significance of adding CRP to the serum albumin levels and lymphocyte counts in patients with resected lung cancer associated with IP.

In the original report by Iida et al., the albumin level, lymphocyte count, and CRP value were included in the CALLY index to reflect the patients’ nutritional status, immune status, and inflammation level, respectively [8]. Although PNI and mGPS are established indices associated with the postoperative prognosis in certain types of cancer, including lung cancer [26, 27], our results indicate that only the CALLY index, and not PNI or mGPS, was associated with the postoperative prognosis. Therefore, all three factors—nutritional status, immune status, and inflammation level—may be important in predicting RFS and OS in patients with resected lung cancer associated with IP.

The cutoff value for the CALLY index varies from study to study. The original report by Iida et al. defined a cutoff value of 5 [8]. For resected esophageal squamous cell carcinoma, gastric cancer, breast cancer, and non-small cell lung cancer, the respective cut-off values have been reported to be 1.7, 6.96, 2.285, and 6.2, respectively, all of which were derived from the analysis of the ROC curve for OS [11–14]. In a study demonstrating the predictive role of the CALLY index for postoperative pneumonia in esophageal cancer, the optimal cut-off value was defined as 3.47 [18]. In contrast, the present study defined the cutoff value as 0.97, suggesting that the optimal cutoff value for the CALLY index varies according to the type of disease and type of cancer. The reason for our lower cutoff value may also lie in the particularly poor nutritional status [28] and more elevated inflammatory status [29] in patients with lung cancer associated with IP. This is partially supported by a comparison of the median CALLY index of our cohort with that reported by Mizota et al. (3.98 [IQR 1.10, 10.93] vs. 7.85 [IQR 2.67, 19.04]) [14]. However, caution is advised with this comparison, as the report by Mizota et al. did not specify the number of lung cancer cases associated with IP in their cohort and statistical analyses were not feasible for this comparison.

IP-AE is a major concern in the postoperative management of resected lung cancer associated with IP because of its high mortality rate (43.9%) [6]. Risk scores for IP-AE have been established in Japan and are widely used [6]. The present study found a potential association between low CALLY and the incidence of IP-AE, although this association was not statistically significant. Matsubara et al. demonstrated a significant association between the controlling nutritional status derived from serum albumin, lymphocyte count, total cholesterol, and IP-AE [30]. These findings suggest that dysregulation of the nutritional status, the immune system, and inflammation might be associated with an increased risk of IP-AE and that the CALLY index could be useful in the preoperative stratification of patients at higher risk of developing IP-AE.

The present study is associated with several limitations. First, it had a retrospective design and was conducted at a single institution. Second, although there were 85 patients in the study, only 20 patients were included in the low CALLY group. Third, the number of OS and RFS events was 45, limiting the number of variables in the multivariate analysis. Fourth, although our study aimed to predict postoperative survival using preoperative variables, data from positron emission tomography/CT were not included because of the lack of data for 36 patients (42.4%). Fifth, our findings are yet to be validated in another cohort of patients. Sixth, the AUC value of 0.577 was relatively low for prediction, which may be due to the small number of events. However, the cutoff value of the CALLY index (0.97) effectively stratified the survival curve and was identified as an independent prognostic factor for RFS and OS in the multivariate analysis. Larger studies with more patients are needed to address these limitations.

This study identified CALLY index as an independent prognostic marker for both RFS and OS in patients with resected lung cancer associated with IP. Furthermore, we found that the postoperative hospital stay was significantly longer, and the incidence of postoperative pneumonia was significantly higher in the low CALLY group than in the high CALLY group. Although studies that include more patients are required, these findings may assist in the postoperative management of patients with resected lung cancer associated with IP, particularly through close follow-up that may lead to the early detection of recurrence, timely treatment intervention, and ultimately improved post-recurrence survival.

## Supplementary Information

Below is the link to the electronic supplementary material.

Supplementary file1 (DOCX 198 KB)

## References

[1] Kumar P, Goldstraw P, Yamada K, Nicholson AG, Wells AU, Hansell DM, et al. Pulmonary fibrosis and lung cancer: risk and benefit analysis of pulmonary resection. J Thorac Cardiovasc Surg. 2003;125:1321–7. 12830051 10.1016/s0022-5223(03)00028-x

[2] Chiyo M, Sekine Y, Iwata T, Tatsumi K, Yasufuku K, Iyoda A, et al. Impact of interstitial lung disease on surgical morbidity and mortality for lung cancer: analyses of short-term and long-term outcomes. J Thorac Cardiovasc Surg. 2003;126:1141–6. 14566260 10.1016/s0022-5223(03)00791-8

[3] Watanabe A, Higami T, Ohori S, Koyanagi T, Nakashima S, Mawatari T. Is lung cancer resection indicated in patients with idiopathic pulmonary fibrosis? J Thorac Cardiovasc Surg. 2008;136:1357–63. 19026828 10.1016/j.jtcvs.2008.07.016

[4] Sato T, Watanabe A, Kondo H, Kanzaki M, Okubo K, Yokoi K, et al. Long-term results and predictors of survival after surgical resection of patients with lung cancer and interstitial lung diseases. J Thorac Cardiovasc Surg. 2015;149:64–9. 25439777 10.1016/j.jtcvs.2014.08.086

[5] Toyokawa G, Hino H, Akamine T, Shimokawa M, Sato M. Surgical outcomes of lung cancer associated with autoimmune disease-related interstitial pneumonia. Gen Thorac Cardiovasc Surg. 2025;3:34. 10.1007/s11748-025-02229-9 PMC13139268 41269532

[6] Sato T, Teramukai S, Kondo H, Watanabe A, Ebina M, Kishi K, et al. Impact and predictors of acute exacerbation of interstitial lung diseases after pulmonary resection for lung cancer. J Thorac Cardiovasc Surg. 2014;147(1604–11):e3. 24267779 10.1016/j.jtcvs.2013.09.050

[7] Iyoda A, Azuma Y, Sakamoto S, Homma S, Sano A. Surgical treatment for patients with idiopathic pulmonary fibrosis and lung cancer: postoperative acute exacerbation of idiopathic pulmonary fibrosis and outcomes. Surg Today. 2022;52:736–44. 34347162 10.1007/s00595-021-02343-0

[8] Iida H, Tani M, Komeda K, Nomi T, Matsushima H, Tanaka S, et al. Superiority of CRP-albumin-lymphocyte index (CALLY index) as a non-invasive prognostic biomarker after hepatectomy for hepatocellular carcinoma. HPB (Oxford). 2022;24:101–15. 34244053 10.1016/j.hpb.2021.06.414

[9] Onodera T, Goseki N, Kosaki G. [Prognostic nutritional index in gastrointestinal surgery of malnourished cancer patients]. Nihon Geka Gakkai Zasshi. 1984;85:1001–5. 6438478

[10] Forrest LM, McMillan DC, McArdle CS, Angerson WJ, Dunlop DJ. Evaluation of cumulative prognostic scores based on the systemic inflammatory response in patients with inoperable non-small-cell lung cancer. Br J Cancer. 2003;89:1028–30. 12966420 10.1038/sj.bjc.6601242 PMC2376960

[11] Feng J, Wang L, Yang X, Chen Q. Clinical significance of preoperative CALLY index for prognostication in patients with esophageal squamous cell carcinoma undergoing surgery. Sci Rep. 2024;14:713. 38184747 10.1038/s41598-023-51109-w PMC10771508

[12] Nakashima K, Haruki K, Kamada T, Takahashi J, Tsunematsu M, Ohdaira H, et al. Usefulness of the C-Reactive Protein (CRP)-Albumin-Lymphocyte (CALLY) Index as a Prognostic Indicator for Patients With Gastric Cancer. Am Surg. 2024;90:2703–9. 38644521 10.1177/00031348241248693

[13] Zhuang J, Wang S, Wang Y, Wu Y, Hu R. Prognostic value of CRP-albumin-lymphocyte (CALLY) index in patients undergoing surgery for breast cancer. Int J Gen Med. 2024;17:997–1005. 38505146 10.2147/IJGM.S447201 PMC10949993

[14] Mizota K, Kinoshita F, Giacomo B, Tokunaga T, Hashinokuchi A, Matsudo K, et al. The prognostic impact of C-reactive protein-albumin-lymphocyte index (Cally Index) in patients with surgically resected non-small-cell lung cancer. Ann Surg Oncol. 2025. 10.1245/s10434-025-18661-3. 41176515 10.1245/s10434-025-18661-3

[15] Yuksel Y, Gur OE, Senirli RT, Yilmaz NDS, Bedel C, Zortuk O, et al. The Effect of CALLY Index and HALP Score on Mortality in Patients with COVID-19. Clin Lab. 2025;71:34. 10.7754/Clin.Lab.2025.250346 41078205

[16] Saridas A, Cetinkaya R. The Prognostic Value of the CALLY Index in Sepsis: A Composite Biomarker Reflecting Inflammation, Nutrition, and Immunity. Diagnostics (Basel). 2025;15:34. 10.3390/diagnostics15081026 PMC12025508 40310418

[17] Zhu L, Jie S, Wu S, Chen Q, Zhang X, Yang W, et al. Ineffective Recanalization and Complications in Patients with Acute Ischemic Stroke Receiving Endovascular Treatment: Predictive Value of the c-Reactive Protein-Albumin-Lymphocyte (CALLY) Index. J Inflamm Res. 2025;18:14649–61. 41146948 10.2147/JIR.S535881 PMC12554279

[18] Xu Z, Chen C, Zhao J, Li C, Zang B, Xiong X. The CALLY index as a predictive tool for postoperative pneumonia in esophageal squamous cell carcinoma: a retrospective cohort study. J Inflamm Res. 2025;18:5463–75. 40291459 10.2147/JIR.S517074 PMC12034254

[19] Travis WD, Costabel U, Hansell DM, King TE Jr, Lynch DA, Nicholson AG, et al. An official American Thoracic Society/European Respiratory Society statement: Update of the international multidisciplinary classification of the idiopathic interstitial pneumonias. Am J Respir Crit Care Med. 2013;188:733–48. 24032382 10.1164/rccm.201308-1483ST PMC5803655

[20] Flaherty KR, King TE Jr., Raghu G, Lynch JP 3rd, Colby TV, Travis WD, et al. Idiopathic interstitial pneumonia: what is the effect of a multidisciplinary approach to diagnosis? Am J Respir Crit Care Med. 2004;170:904–10. 15256390 10.1164/rccm.200402-147OC

[21] Raghu G, Collard HR, Egan JJ, Martinez FJ, Behr J, Brown KK, et al. An official ATS/ERS/JRS/ALAT statement: idiopathic pulmonary fibrosis: evidence-based guidelines for diagnosis and management. Am J Respir Crit Care Med. 2011;183:788–824. 21471066 10.1164/rccm.2009-040GL PMC5450933

[22] Sato T, Kondo H, Watanabe A, Nakajima J, Niwa H, Horio H, et al. A simple risk scoring system for predicting acute exacerbation of interstitial pneumonia after pulmonary resection in lung cancer patients. Gen Thorac Cardiovasc Surg. 2015;63:164–72. 25352311 10.1007/s11748-014-0487-6

[23] Ryerson CJ, Vittinghoff E, Ley B, Lee JS, Mooney JJ, Jones KD, et al. Predicting survival across chronic interstitial lung disease: the ILD-GAP model. Chest. 2014;145:723–8. 24114524 10.1378/chest.13-1474

[24] Fujii H, Yamada Y, Iihara H, Kobayashi R, Suzuki A. Anamorelin in the management of cancer cachexia: clinical efficacy, challenges, and future directions. Anticancer Res. 2025;45:865–81. 40037850 10.21873/anticanres.17475

[25] Richeldi L, Azuma A, Cottin V, Kreuter M, Maher TM, Martinez FJ, et al. Nerandomilast in patients with idiopathic pulmonary fibrosis. N Engl J Med. 2025;392:2193–202. 40387033 10.1056/NEJMoa2414108

[26] Shoji F, Morodomi Y, Akamine T, Takamori S, Katsura M, Takada K, et al. Predictive impact for postoperative recurrence using the preoperative prognostic nutritional index in pathological stage I non-small cell lung cancer. Lung Cancer. 2016;98:15–21. 27393501 10.1016/j.lungcan.2016.05.010

[27] Pinato DJ, Shiner RJ, Seckl MJ, Stebbing J, Sharma R, Mauri FA. Prognostic performance of inflammation-based prognostic indices in primary operable non-small cell lung cancer. Br J Cancer. 2014;110:1930–5. 24667648 10.1038/bjc.2014.145 PMC3992503

[28] Faverio P, Bocchino M, Caminati A, Fumagalli A, Gasbarra M, Iovino P, et al. Nutrition in patients with idiopathic pulmonary fibrosis: critical issues analysis and future research directions. Nutrients. 2020. 10.3390/nu12041131. 32316662 10.3390/nu12041131 PMC7231241

[29] Cottin V, Valenzuela C. C-reactive protein as a candidate biomarker in fibrotic interstitial lung disease. Respirology. 2024;29:195–8. 38296837 10.1111/resp.14666

[30] Matsubara T, Shimokawa M, Wakasu S, Haro A, Yamaguchi M, Hamatake M. Controlling nutritional status predicts postoperative survival and acute exacerbation in resected non-small cell lung cancer with interstitial lung disease. Lung Cancer. 2025;205:108591. 40435687 10.1016/j.lungcan.2025.108591

