# Supplementary material for: Prognostic impact of the C-reactive protein–albumin–lymphocyte index in patients with resected lung cancer associated with interstitial pneumonia
Source: Surg Today. 2026 Mar 14;56(8):1570–8. doi: 10.1007/s00595-026-03250-y (PMC13379499; doi:10.1007/s00595-026-03250-y)
Supplement: Supplementary file 1 — Supplementary file1 (DOCX 198 KB) [file 595_2026_3250_MOESM1_ESM.docx]

**Supplementary Fig. 1** Receiver operating characteristic curve for the ability of the CALLY index to predict the overall survival following surgery. AUC, area under the curves; CALLY, C-reactive protein–albumin–lymphocyte count.


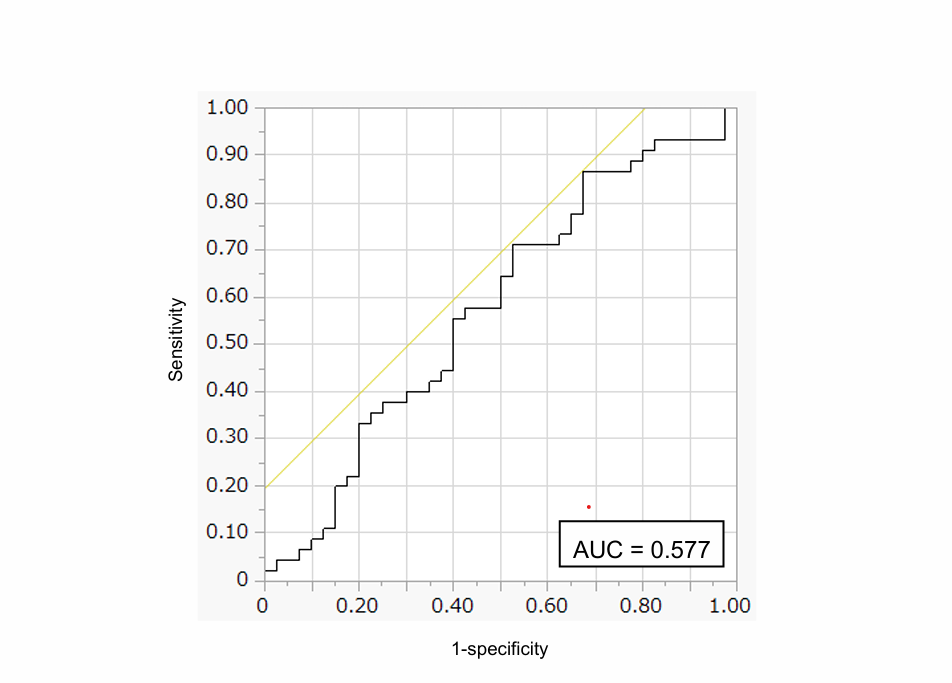


**Supplementary Fig. 2** Survival following surgery according to the PNI and mGPS. (a) Receiver operating characteristic curve for the ability of the PNI to predict the overall survival following surgery. (b) Comparison of the overall survival between the low and high PNI groups. (c) Comparison of the overall survival between the group with mGPS = 0 and the group with mGPS = 1 or 2. AUC, area under the curves; mGPS, modified Glasgow prognostic score; PNI, prognostic nutritional index.


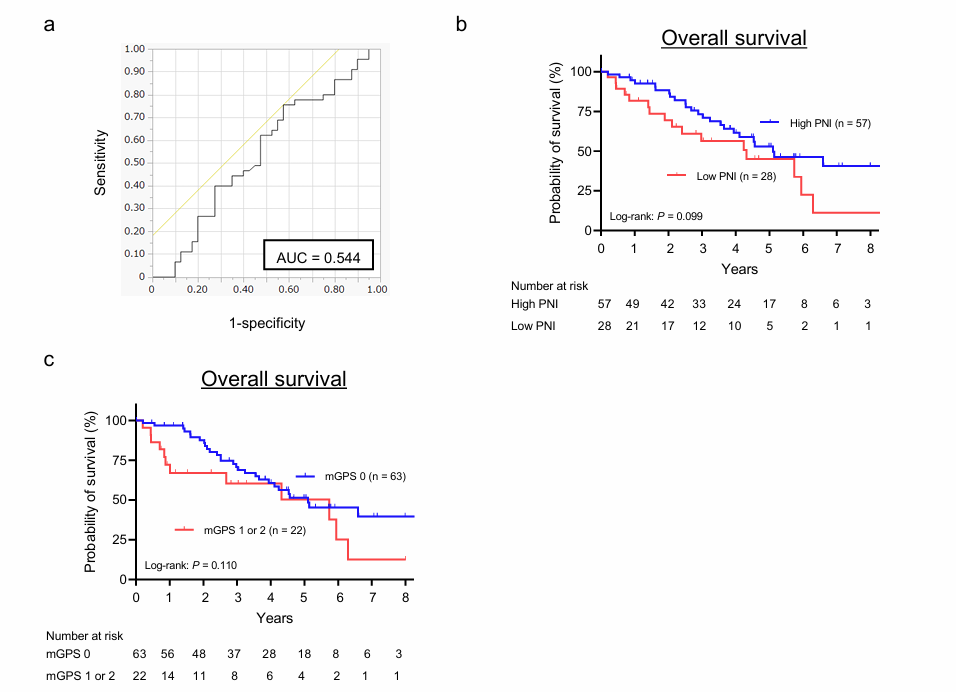


**Supplementary Fig. 3** Survival following surgery according to CRP and albumin. (a) Comparison of the overall survival between the low and high CRP groups. (b) Comparison of the overall survival between the low and high albumin groups. CRP, C-reactive protein.

**Supplementary Figure 3.**

Overall survival

a

b

Overall survival
